# Utilization of Mechanically Recycled Carbon Fibers in Vinyl Ester Composites

**DOI:** 10.3390/polym15041016

**Published:** 2023-02-17

**Authors:** Khaled AlHarmoodi, Amir Hussain Idrisi, Abdel-Hamid Ismail Mourad, Basim Abu-Jdayil

**Affiliations:** 1 Department of Mechanical and Aerospace Engineering, United Arab Emirates University, Al Ain 15551, United Arab Emirates; 2 Mechanical Engineering-Engineering Mechanics, Michigan Technological University, Houghton 49931, MI, USA; 3 National Water and Energy Centre, United Arab Emirates University, Al Ain 15551, United Arab Emirates; 4 Department of Chemical Engineering, United Arab Emirates University, Al Ain 15551, United Arab Emirates

**Keywords:** carbon fibers, vinyl ester, polymer composites, recycling

## Abstract

As we enter the twenty-first century, the aviation sector is expected to thrive as flying becomes the primary mode of transportation between states or nations. With such a demand, there is a corresponding need to manufacture aircraft components. The study focused on recycling carbon fiber composites received from the STRATA company, which were cut-off/waste material generated during the manufacture of airplane components. The cut-offs were then reduced to powder form using a standard face milling machine in three sizes (90, 150, and 250 µm). After, the powder was utilized to fabricate vinyl ester composites with four weight percentages (10%, 20%, 30%, and 40%). The results demonstrate that the tensile strength of all composites had risen by 30.2%, 21.3%, and 17.6% for 90, 150, and 250 µm respective with the addition of 20 wt% of reinforcement. Furthermore, subsequently decreased with the additional reinforcement for all particle sizes. The compressive strength increased by 30% from 187.5 MPa to 244 MPa with 10 wt% of recycled carbon powder composite of 90μm particle size. However, samples prepared with 150 μm and 250 μm fiber size show approximately 17% and 1% increase in the compression strength with the addition of 10wt% of recycled carbon powder. A similar trend was observed for the flexural strength with an highest increase of 9% for 90 µm particle size with addition of 20 wt% reinforcement. Nonetheless, the SEM images revealed that the fiber–matrix bonding was weak, proved through the clean pullout fibers at the fracture surfaces.

## 1. Introduction

**Figure 1:**
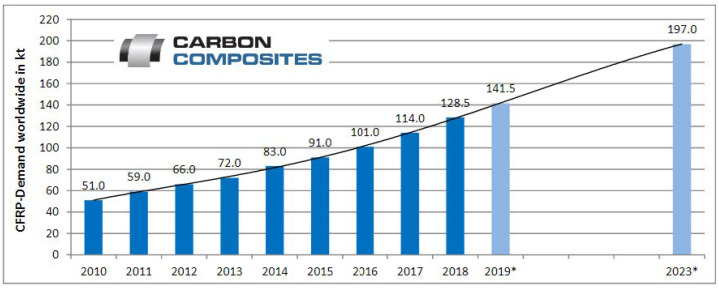
CFRP demand **[12]**, * Estimations; 09/2019.

Reinforced composites are formed by stacking layers of reinforcing fibers (such as carbon, glass, and Kevlar) to a certain weight percentage and then being impregnated in polymers, either thermoplastics or thermosets. Thermoplastic polymers can be categorized as crystalline, semi-crystalline, or amorphous. The production of thermoplastic polymers has increased dramatically in recent decades due to their suitability for industrial applications. For instance, thermoplastic polymers are found to possess low density and corrosion resistance while being cost-effective and durable. Textiles, food packaging, medical materials, and agricultural pipes are examples of the diverse applications that these polymers have shown [1,2,3,4,5,6]. The global demand for carbon fibers has shown steady growth since the general economic recession of 2009 at 26.5 kt, as shown in Figure 1. The annual growth rates increased to over 20% after 2009 and reduced over the following years to a growth rate of 6.9% in 2013. In 2014, the growth rate increased to 14% and leveled at 9.4% in 2015. This translates to roughly 58 kt, and the market is estimated at 2.15 billion USD compared to 1.98 billion USD, with a growth of 8.6% in 2015. On the other hand, the carbon fiber reinforced plastics (CFRP) market has seen a growth rate of around 10% from 2014 to 2015, reaching 91 kt and a revenue of 11.6 billion USD [7]. This strong growth continued in 2016, reaching 101 kt and a revenue of 13.23 billion USD, and in 2017 reaching 114 kt and a revenue of 14.73 billion USD [8].

Similarly, in 2018, a growth of 12.7% was recorded, with the global demand for CFRP reaching a value of 128.5 kt and sales of 16.31 billion USD. For 2019, a demand of 141 kt and a turnover of approximately 17.88 billion USD is estimated and mounting to a demand of 197 kt in 2023 [9]. Alongside this demand, there is also the generated waste, estimated to be 3 kt yearly. Additionally, around 6000–8000 aircraft will reach their end-of-service in 2030 [10]. Studies have shown that up to 40% of the CFRC waste is produced during manufacturing [11], with a forecast to reach 20 kt annually by 2025 [12]. With the increased demand for fiber-reinforced plastics, an alternative route for end-of-life parts is becoming critical. While landfills might seem attractive, it is the least preferred option due to the massive environmental impact it poses. Therefore, ecological legislations are becoming very restrictive, with some countries such as Germany already having passed laws prohibiting the disposal of composites at landfills [13]. These legislations have further pushed recycling efforts encompassing mechanical, pyrolysis, and other thermal processes and solvolysis approaches. As a result, the mere steps have grown to industrial-scale plants such as ELG Carbon Fibre Ltd. in the United Kingdom [14]. Other companies have used chemical breakdowns to recover the fiber reinforcement, such as Innoveox Inc. in France [15]. In the emirate of Abu Dhabi in particular, the Environmental Agency has stated in their 2021 annual report that the industrial and commercial wastes amounted to 35.35% of the non-hazardous waste generated (9.77 million tons), of which only 31.5% was recycled, 66.1% was sent to dumpsites, and 1.9% was sent to landfills. As such, the agency has set a short-term target to increase total non-hazardous solid waste diversion to 39% by 2025. Keeping this in mind, recycling CFRC is essential to minimize any adverse environmental effects.

Recycling processes are broadly grouped into pyrolysis, solvolysis, and mechanical. The most popular approach is pyrolysis, which involves heating the composites at high temperatures in the absence of oxygen to burn away the resin and get the carbon fibers. The gases emitted during the incineration process depend on the resin used to form the composite, and the organic component combination can be recovered via the condensation process. Meyer et al. [16] conducted preliminary research to investigate the influence of the heating rate, pyrolysis temperature, and other parameters on the mechanical characteristics of produced carbon fibers versus those of virgin fibers. The test was carried out on a batch of carbon fiber reinforced composites in a gas oven at pyrolysis temperatures of 700 °C, 900 °C, 1100 °C, and 1300 °C. The layer of degraded epoxy thinned out at higher temperatures and wore away at 1300 °C. This pyrolytic carbon layer is critical because any remnant on the carbon fiber surface will impair mechanical and electrical qualities. Further to that, it should be noted that the carbon became significantly activated as a result of the high temperatures.

The second most frequent approach is solvolysis, which involves chemically treating the composite with an acid or an alkali to separate the resin and release the carbon fibers. Keith et al. [17] investigated the influence of a supercritical solvent on the separation of resin and carbon fiber. They discovered that a supercritical combination of acetone and water generated a 98% deterioration in 2 h at temperatures and pressures of 320 °C and 170 bar, respectively. Unfortunately, the resultant solution of the solvolysis process was exceedingly dangerous, posing a threat to people’s and the environment’s health.

However, little work has been conducted in mechanical recycling due to the limited use of recovered fibers in the third category. The viability of reusing discarded short fibers in the creation of new thermoplastic composites was examined by Kouparitas et al. [18]. They employed glass fibers from recycled glass–polyester composites and carbon and aramid fibers from epoxy-based composites. Composite samples were chopped into smaller pieces and processed into fine powder. Following the preparation of the samples, a particular fibrous fraction was chosen and analyzed in terms of the fiber length and residual matrix composition. The fiber material was integrated into virgin composites to create novel thermoplastic composites. Recycled glass fibers were combined with polypropylene, recycled carbon fibers with an ionomer, and recycled aramid fibers with an ionomer to form new composites. The mechanical recycling approach was shown to have no negative impact on the mechanical performance of the new composite, which has been attributed to fiber length preservation, fiber dispersion mechanism, and fiber matrix adhesion.

Takashi et al. [19] investigated the influence of recycled carbon fibers on the tensile and flexural strength of the composite. The recycled composite was created using thermoplastic resins such as polypropylene (PP) and acrylonitrile–butadiene–styrene (ABS). The method began with crushing the CFRP into 1cm squares, and the thermoplastics were dried for 8 h at 80 °C. Then, carbon fiber reinforced thermoplastics (CFRTP) pellets were created using a two-axis pelletizing machine. Several pellet samples were made with various fiber volume percentages of 7, 15, 24, and 30%. Subsequently, the pellets were used to create samples for bending, tensile, and impact tests utilizing an injection molding machine. The results revealed that the maximum flexural strength was obtained with a 24% fiber volume percentage of ABS.

Ogi et al. [20] did a similar study where they employed scrap carbon fiber reinforced acrylonitrile–butadiene–styrene (ABS) composites that were size reduced via a crushing machine. The samples were created by injecting ABS resin and crushed carbon fiber waste into a mold. The crushed CFRP fragments measured 3.4 × 0.4 mm^2^ on average. A portion of the CFRP pieces was further crushed by milling, resulting in powder form with particles ranging in size from 1 to 10 µm. Throughout the experiment, nine distinct compounds with variable percentages of weight/size of CFRP pieces were created and examined for mechanical properties. The tensile and flexural strengths increased with increasing CFRP content, whereas the tensile and flexural modulus increased linearly with increasing CFRP content, following the law of mixing. Milled CFRP produced significantly lower strength and modulus when compared to another sample with a similar CFRP composition. The disparity in findings was ascribed to the fiber length being less than 10 µm (below the crucial length).

Okayasu et al. [21] investigated the tensile properties of recycled carbon fiber-reinforced plastic mixed with various resins, such as unsaturated polyester and epoxy. A commercial CFRP sheet with a volume fraction of 60% carbon fiber (Nippon Steel and Sumikin Materials Co. Ltd.) was ground to create milled fragments of average dimensions of 0.1 mm × 0.007 mm and chopped flakes with average dimensions of 30 mm × 2 mm. The fragmented CFRP was mixed with the resins at four predefined fractions (0, 10, 20, and 30 wt%) and poured into a rectangular mold of dimensions 10 mm × 50 mm × 1 mm. The tensile strength of rCFRP was found to increase with the incremental addition of the chopped CFRP but decreased with an increasing fraction of milled CFRP. It was inferred that the tensile strength depends on the size of the CFRP fragments and the proportion, while it didn’t exhibit any noticeable dependency on the type of resin used. As for the fracture strain, a low fracture strain was determined for rCFRP samples fabricated with chopped CFRP, in contrast to those made with milled CFRP.

Mamanpush et al. [22] looked into the possibility of developing a heterogenous composite made of thermoset and thermoplastic recycled carbon fiber composites (CFC). In their study, the researchers used epoxy/CFC and VE/CFC scraps provided by Zoltek Corporation (Bridgeton, MO, USA) and PEEK/CFC provided by Triumph Composites (Spokane, WA, USA). A hammer mill with a 25.4 mm screen size was used to mechanically reduce the size of the scraps to those of size less than 3.4mm and less than 1.2 mm. These were then used to fabricate samples in a mold of dimensions 355.6 × 355.6 × 6.35 mm using a compression molding machine with a process parameter of 360°–400 °C. All samples were conditioned for 24 h at a temperature of 23 °C and a relative humidity of 50%. The addition of 15% of VE/CFC to the PEEK/CFC improved the composite’s flexural modulus of elasticity by roughly a factor of 2 at 400 °C platen temperature. On the other hand, at 400 °C, adding 15% and 30% of epoxy/CFC to the PEEK/CFC enhanced the flexural MOE of the composite by 20% and 100%, respectively. Results indicate that rCFCs with smaller particle sizes improved the flexural properties and reduced the tensile properties of heterogeneous composites.

Given the preceding literature, the work on composites recycling requires additional attention, particularly in aircraft composite waste. This research will primarily concentrate on producing composites for lower-grade applications using mechanically recycled carbon fiber-reinforced polymer resins. Cured prepreg waste was collected, mechanically recycled, and combined with vinyl ester resins to create new composites. These composites were then subject to characterization and assessment, with the main outcome being attributed to the reduction in environmental impact where ecological legislations are becoming very restrictive, with some countries already passing laws prohibiting the disposal of composites at landfills. Further to that, the researchers aim to contribute to the development of a new method to convert industrial waste into valuable goods of varying classes based on chemical, physical, and mechanical qualities obtained.

## 2. Materials and Methods

### 2.1. Vinyl Ester

A commercial grade Vinyl Ester (OCPOL 800VE) was procured from Al-Khowahir Chemicals, located in Sharjah. The liquid properties of the resin are mentioned in Table 1.

### 2.2. Carbon Fibers

STRATA, being one the leading entities in the aerospace industry located in the Al Ain, Abu Dhabi, United Arab Emirates, is heavily focused on the utilization of carbon fiber composites in the production of their components. Therefore, the team has focused its attention on the usage of these materials. The properties as provided are detailed in Table 2.

According to the manufacturer, these composite panels were fabricated via the curing carbon fiber layers in a stacked-up arrangement. The tensile and flexural strength of the panel was 602 MPa and 45.4 MPa, respectively. As for the cured prepregs supplied by STRATA, they were in panels of varying sizes and types. Therefore, the panel had to be broken down to the extent that allowed its incorporation into the samples to be fabricated. While a band saw was initially used to reduce the size of the material mechanically, the attempt was deemed unsuccessful owing to the panel’s superior mechanical strength. As such, switching to a conventional face milling machine (Optimum Maschinen, Hallstadt, Germany) seemed more suitable and efficient.

### 2.3. Sample Preparation

**Figure 2:**
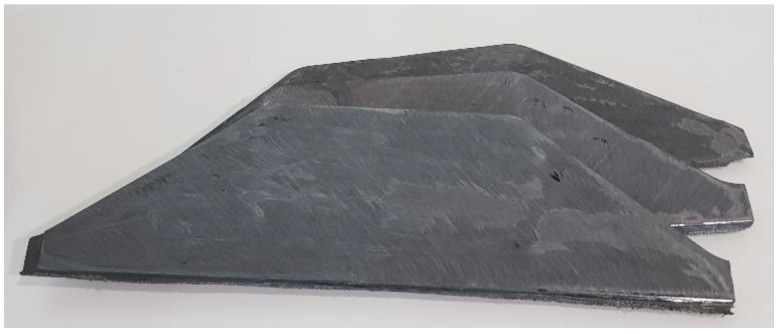
Carbon fiber composite panels.

The mechanical properties of these carbon fiber panels were obtained from the STRATA company. The composite’s tensile, flexural, and Interlaminar Shear Strength were 601.8 MPa, 45.4 MPa, and 73.8 MPa, respectively. Since the cured prepregs were in the form of panels varying in size, as shown in Figure 2, a mechanical size reduction process needed to be undertaken before incorporation into the samples was to be completed.

**Figure 3:**
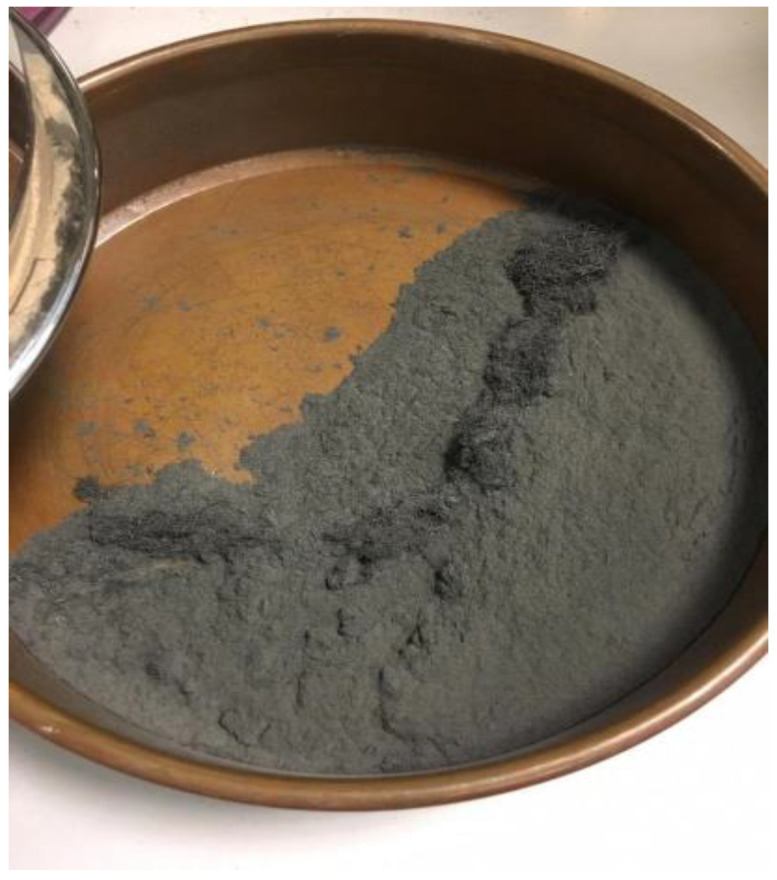
Sieved carbon fiber powder.

The utilization of the conventional face milling machine yielded fine carbon fiber sizes in the range of 400 μm as. This was then sieved off to the required size levels (90 μm, 250 μm), as shown in Figure 3.

The powdered carbon fiber composite which was porous at the time was mixed with the vinyl ester resin (Al Khowahir Chemicals Mat. Trading L.L.C, Sharjah, UAE) in selected proportions (10, 20, 30, and 40 % wt) at a proper constant rotational speed for a specific time to ensure uniform mixing. The powdered carbon fiber composite was mixed with the vinyl ester resin at a proper constant rotational speed. The mixing time was selected on a trial basis to assess the homogeneity of the fibers within the sample. Afterward, a 5-min mixing time was selected and deemed sufficient to ensure uniform mixing. Other researchers have used a spectrum of mixing time in the literature, using either manual stirring or via a magnetic stirrer [23,24,25,26]. The hardener (Al Khowahir Chemicals Mat. Trading L.L.C, Sharjah, UAE) was added to the mixture and stirred for an additional 1 min before being placed in a vacuum chamber for 10 min. The chamber is used to help reduce the possibility of voids formation within the sample [23,25]. In addition, the overall mixture preparation time was not more than 16 min, which is below the limit of the gel time as shown in Table 1. The homogeneity can also be verified with the results discussed in tensile, compressive and flexural test, discussed in upcoming sections, in which the standard deviation of the properties from the average value is acceptable for such types of composites. After the air entrapment within the mixture is minimized, it is poured into the waxed different prepared molds and left to cure for 24 h in ambient conditions. The samples’ molds shall be fabricated from stainless steel according to ASTM standards for all the designated tests. The following section details the tests conducted, the associated testing equipment, the sample preparation procedure, and the relevant parameters.

#### 2.3.1. Water Absorption

**Figure 4:**
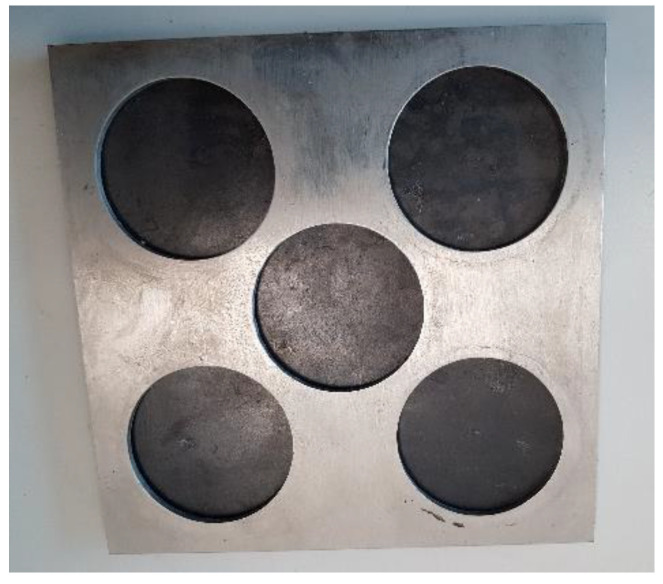
Water retention in specimen’s mold.

The water absorption test was performed to understand the likelihood of water absorption in the different fabricated samples and its suitability for the envisioned application. The samples were fabricated according to the ASTM standard ASTM D570-98. The mold used to fabricate the samples is shown in Figure 4.

**Figure 5:**
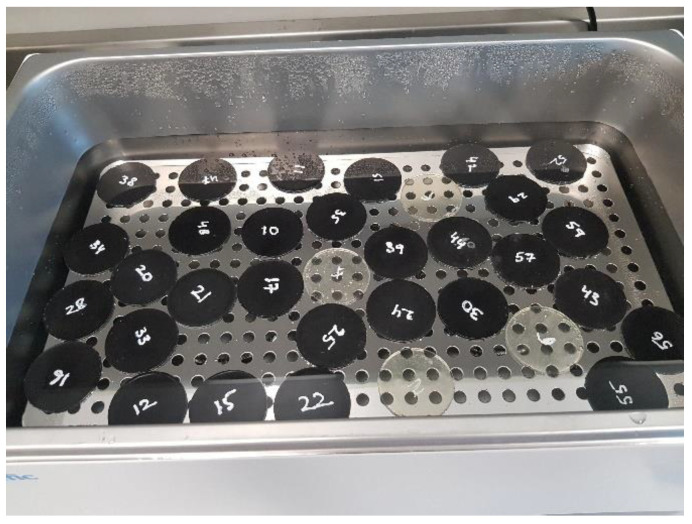
Samples in a water bath.

Firstly, the initial weight of the samples was measured before the immersion in the water. After measuring the weight, samples were immersed in water at room temperature (23 °C) and 50 °C, shown in Figure 5. Initially, the weighing of samples was conducted at 24 h intervals, and then it was extended to week intervals to have a noticeable weight change. The percentage mass change in the composite samples (*M*) was to be calculated using Equation (1):

(1) $$M=\frac{M_{t}-M_{o}}{M_{o}}100\left[\%\right],$$

where:

*M*:  percentage mass change;
*M_t_*:  mass of the sample after a given immersion time (g); and
*M_o_*:  original mass of the sample (g);

#### 2.3.2. Tensile

**Figure 6:**
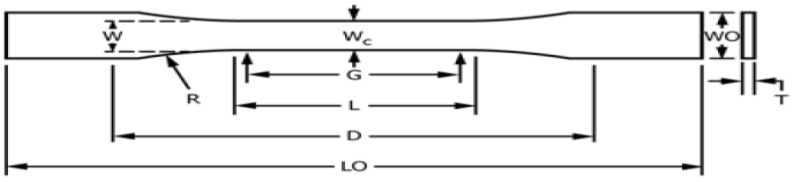
Tensile sample dimensions, where: W: 13 ±0.5 mm; L: 57 ±0.5 mm; WO: 19 ±6.4 mm; LO: 165 mm; G: 50 ± 0.25 mm; D: 115 ±5 mm; R: 76 ±1 mm; and T: 3.2 ±0.4 mm.

**Figure 7:**
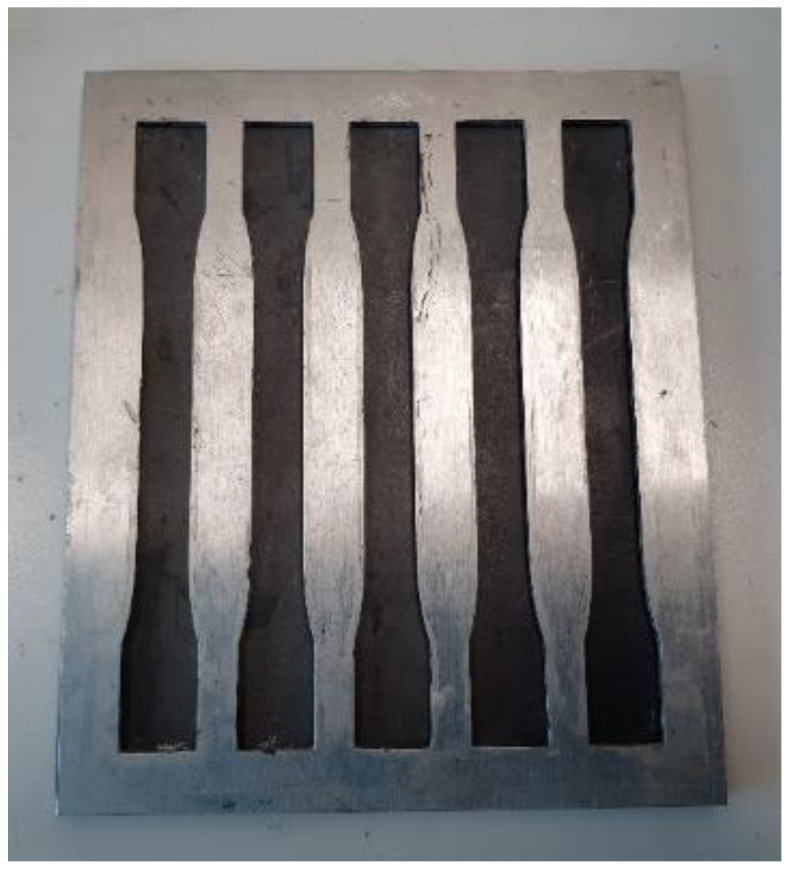
Tensile specimen’s mold.

The tensile strength of the composites was determined for different samples fabricated according to the tensile test method standard ASTM D-3039 (Standard Test Method for Tensile Properties of Polymer Matrix Composite Materials). The tensile test was carried out at room temperature (23 ℃) and 50% humidity with a crosshead displacement of 2 mm/min using the MTS Universal Testing Machine (MTS system corporation, Eden Prairie, MN, USA) with a 50 kN load cell. The dimensions used are illustrated in Figure 6, and the mold in Figure 7.

**Figure 8:**
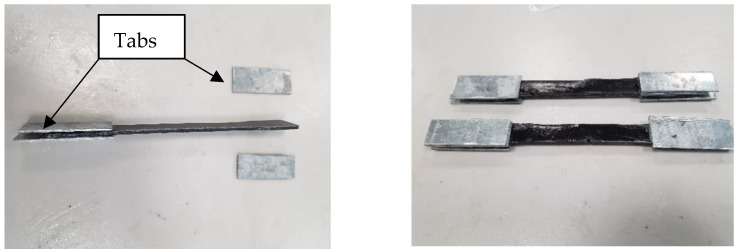
Aluminum tabs glued to tensile samples.

Due to the setup of the tensile machine, the thickness of the fabricated samples’ shoulders had to be altered to fit in the mechanical jaws and act as a stress absorber preventing crack initiations at the points of contact. As such, rectangular-shaped aluminum shoulder tabs of dimensions 50 mm × 20 mm × 2 mm were attached to the sample using adhesive, as shown in Figure 8.

#### 2.3.3. Compression

**Figure 9:**
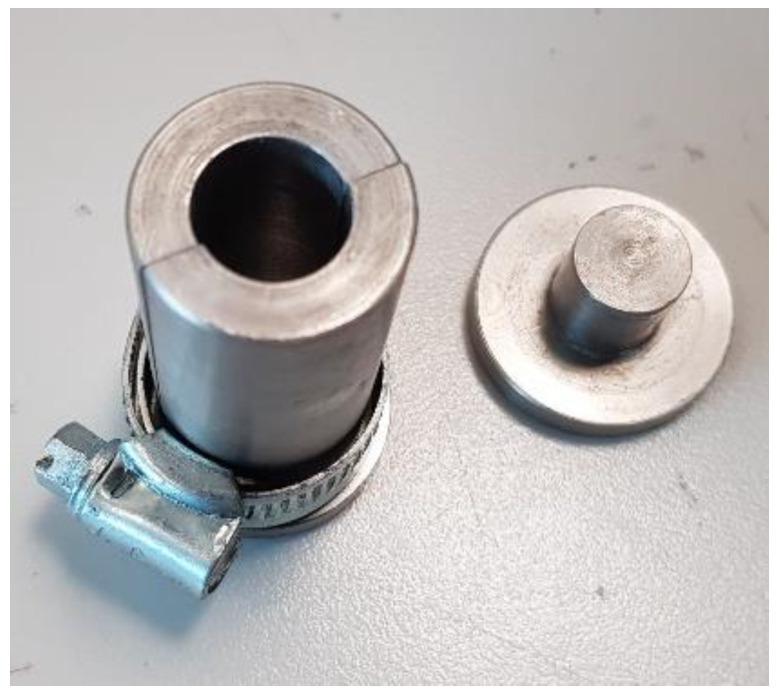
Compression of specimen’s mold.

**Figure 10:**
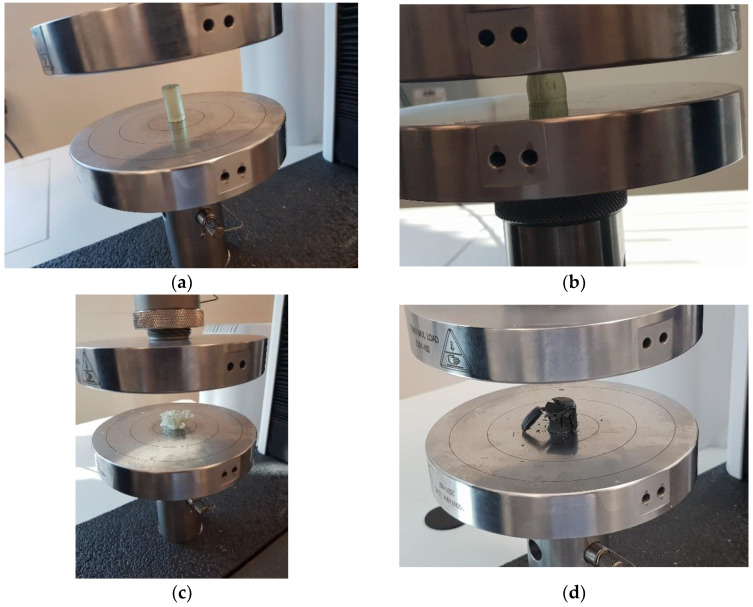
Testing of compression samples.

The compressive strength of the composites was determined for different samples fabricated according to the compression test method standard ASTM D695-15. The mold used is illustrated in Figure 9. The tests were carried out using the MTS Universal Testing Machine with a 50 kN load cell at a crosshead displacement of 5 mm/min, as shown in Figure 10.

#### 2.3.4. Flexural

**Figure 11:**
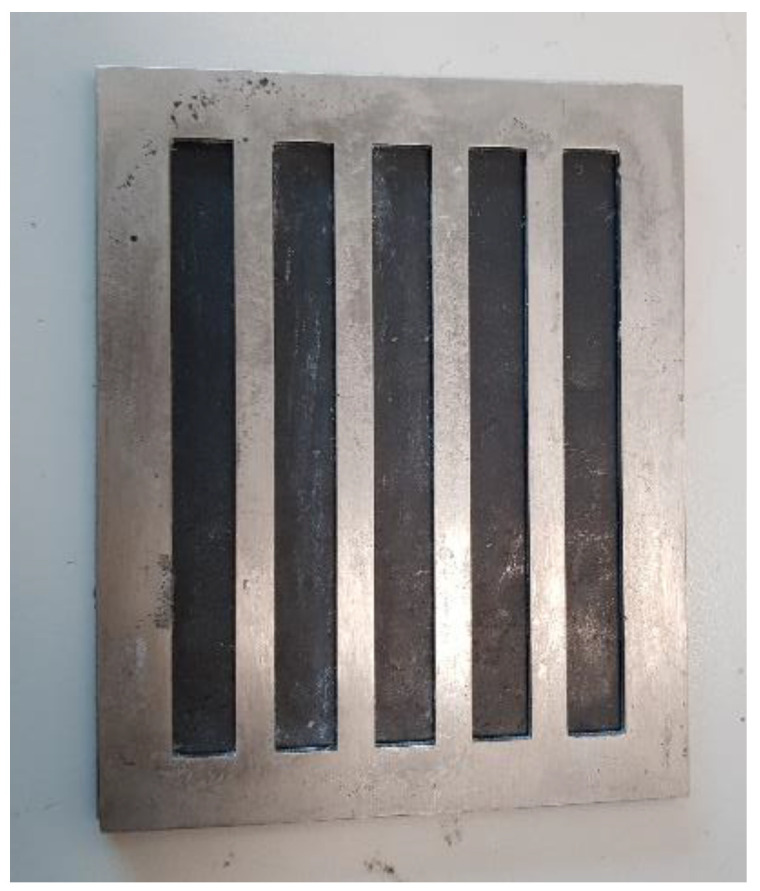
Flexural specimen’s mold.

The flexural strength and modulus of the composites were determined using the 3-point bend test method in accordance with ASTM D790 (Standard Test Methods for Flexural Properties of Unreinforced and Reinforced Plastics and Electrical Insulating Materials). Flexural samples of dimensions 127 mm × 12.7 mm with an average thickness of 3 mm were prepared using a designated mold, as shown in Figure 11.

**Figure 12:**
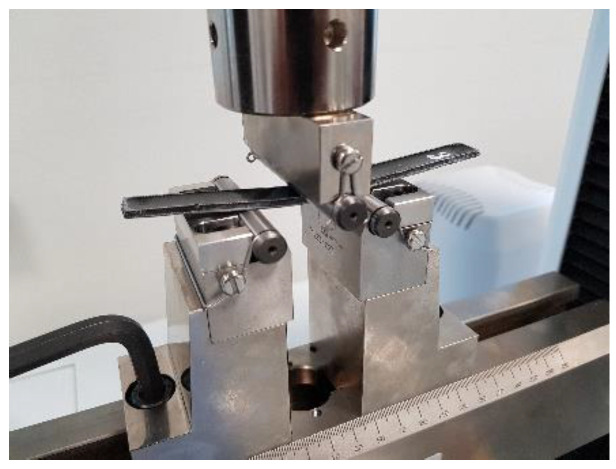
Testing of flexural samples.

The specimens were tested with a span length appropriate to the specimen’s average thickness and a crosshead displacement rate of 1 mm/min, as shown in Figure 12.

The flexural strength (σf) and the flexural modulus (EB) of the composite were calculated as per Equations (2) and (3):

(2) $${}_{f}=\frac{3\mathrm{PL}}{2{\;\mathrm{bd}}^{2}},$$

(3) $$E_{B}=\frac{L^{3}m}{4{\mathrm{bd}}^{3}}$$

where:

$\sigma_{f}$:  stress in the outer fibers at the midpoint (MPa);
P:  load at a given point on the load-deflection curve (N);
$E_{B}$: modulus of elasticity in bending (MPa);
L:  support span (mm);
b: width of beam tested (mm);
d:  depth of beam tested (mm); and
m:  slope of tangent to the initial straight-line portion of the load–deflection curve (N/mm of deflection).

#### 2.3.5. Thermal Conductivity

**Figure 13:**
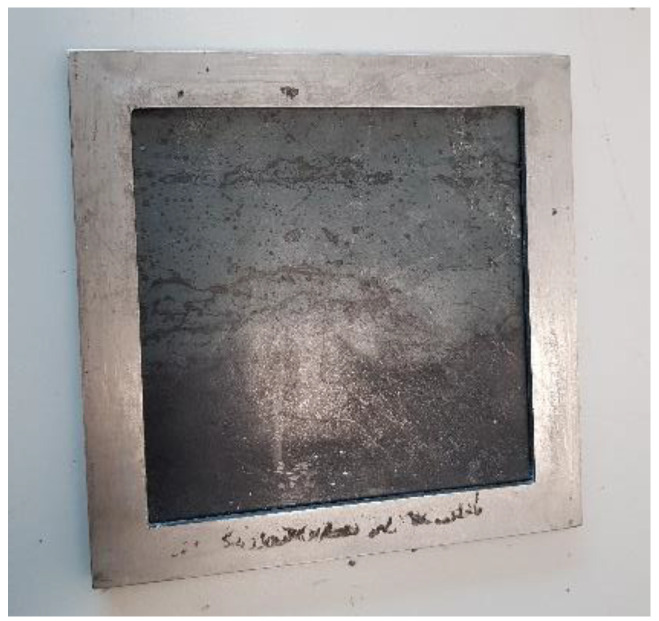
Thermal conductivity of specimen’s mold.

The thermal conductivity of the composites was determined for different samples fabricated according to the test method standard ASTM D5930-17. The mold used is illustrated in Figure 13.

#### 2.3.6. DSC

Differential Scanning Calorimetry (DSC) tests were performed on control and conditioned cured samples to determine the glass transition temperature using TA Instrument Q200 series (TA-Instruments, New Castle, DE, USA).

#### 2.3.7. SEM

The fracture surfaces of the tensile samples were examined for visible signs of fiber/resin degradation due to the long-term exposure using a Scanning Electron Microscope (SEM) on control and exposed samples for both the compacted and non-compacted. The scanning electron microscope was JEOL JSM-5600 (JOEL Ltd., Tokyo, Japan).

The samples were cut into a 1 cm × 1 cm size and fixed vertically on the SEM specimen stub using double-sided conductive carbon tape and were coated with a gold layer using JEOL JFC-1200 Fine Coater (JOEL Ltd., Tokyo, Japan).

## 3. Results

### 3.1. Water Retention

**Figure 14:**
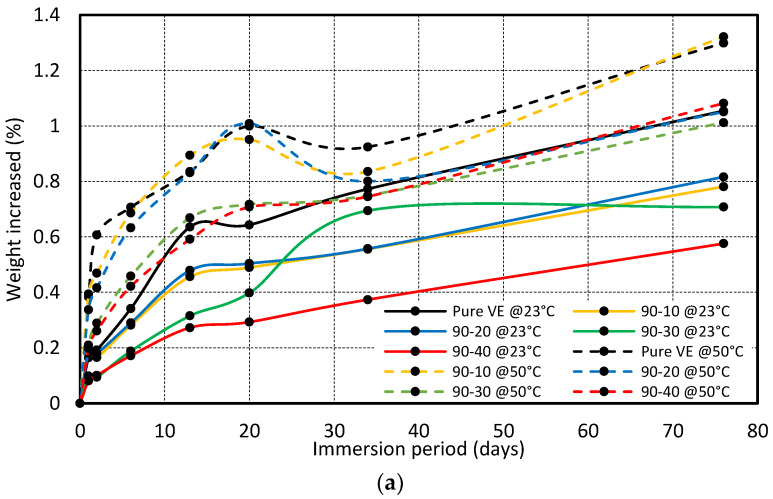

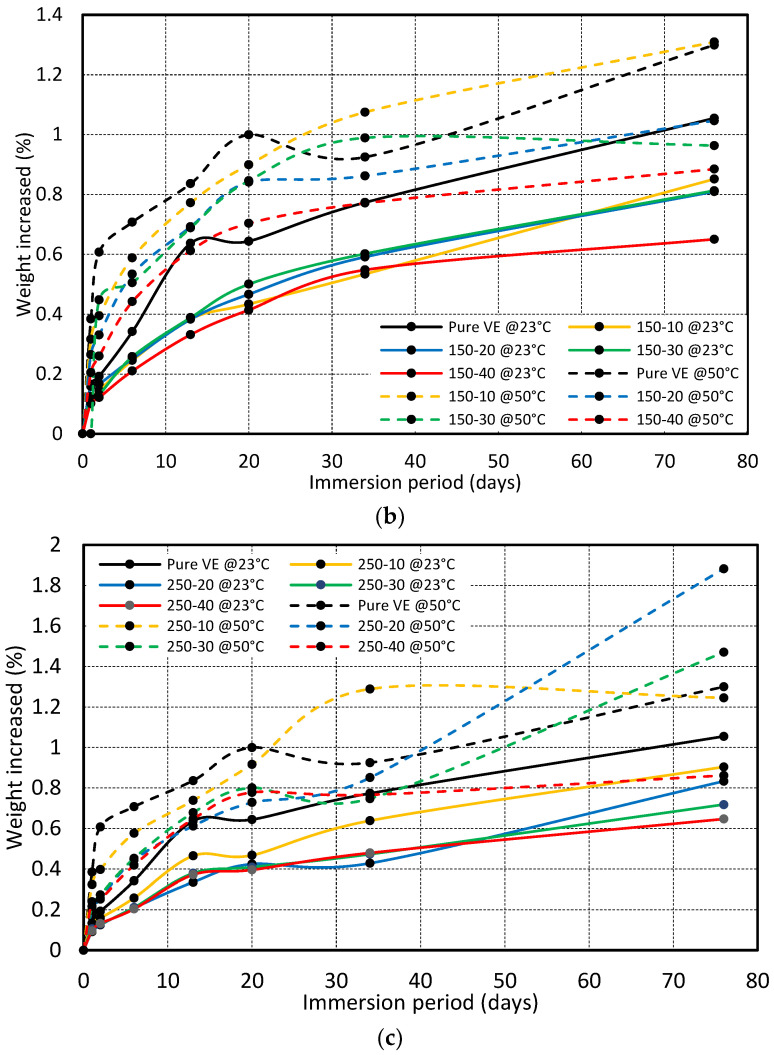
Variation in weight of sample immersed in water at 23 °C and 50 °C fabricated with different weight percentages (0–40wt%) and fiber sizes (**a**) 90 µm, (**b**) 150 µm, and (**c**) 250 µm.

The weight of specimens was taken before immersion into the water at different temperatures. Specimens were removed from the water tank at different time intervals to measure the weight change and immersed again for the subsequent measurement. Figure 14 shows the percentage change in the weight of the specimen after various periods of immersion. The effect of reinforcement percentage on the water absorption is discussed in Figure 14a–c for the particle size of 90 µm, 150 µm, and 250 µm, respectively. It is evident that the water abortion is more for the immersion at 50 °C compared to 23 °C, regardless of the immersion time, reinforcement percentage, and size of the particle

Figure 14 shows that the maximum water absorption was 1.05% and 1.3% for the pure vinyl ester composite at 23 °C and 50 °C after the immersion of 76 days. The amount of water absorbed was reduced with the addition of reinforcement. The weight increase rate was almost the same for the composite prepared with reinforcement sizes of 90 µm and 150 µm, as indicated in Figure 14a,b. The water absorption was high in the composite prepared with a fiber size of 250 µm compared to 90 µm and 150 µm. This could be due to the porosities and cavities developed in the composite during fabrication.

The results show that water absorption increases with exposure time, and the overall rise in weight is caused by water absorption via diffusive and capillary mechanisms. Diffusion is a crucial mechanism that allows moisture to infiltrate polymeric composites and is recognized as a matrix-dominated phenomenon in which water is primarily dispersed in the matrix [27,28,29,30]. Moisture is pulled into voids and microcracks at the fiber/matrix interfaces via the capillary process, where the cracks serve as a transport mechanism for moisture penetration [31,32]. This was also witnessed by Dona et al., where the total water uptake was attributed to the two mechanisms, Fickian diffusion in the resin matrix, and capillary flow in the voids. In their experiment, the Fickian diffusion had a small but significant contribution to the total moisture uptake, about 0.1% for all panels. A substantial fraction of the total water uptake was attributed to the capillary filling mechanism, which was observed to be rapid during the initial regime and decreased until saturation. Nonetheless, the researcher pointed out other factors that influenced the experimental process, such as the repeated taking out and dipping of the samples, which led to variations in the pressure and volume of the trapped air bubbles in the capillary voids, hence the amount of absorbed water [33].

### 3.2. Tensile Test

**Figure 15:**
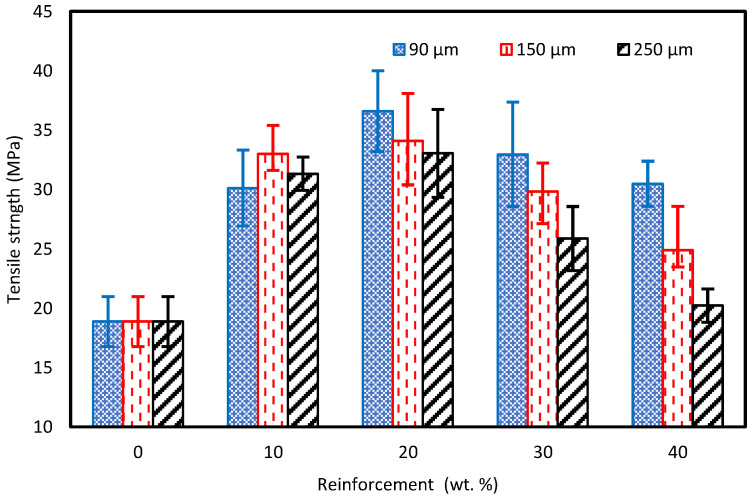
Tensile strength for carbon fiber-reinforced VE composites.

The tensile test on the prepared samples was performed at room temperature (23 ℃) at 50% humidity using an Instron Universal Testing Machine with a 50 kN load cell at a crosshead displacement of 2 mm/min (ASTM D-3039). For the pure vinyl ester (VE) sample, a tensile strength of 28.12 MPa was obtained. At the same time, adding cured carbon fiber powder to the matrix resulted in a general tensile strength increase of up to 20 wt%, followed by a decrease with the further incremental addition of reinforcement. This is illustrated in Figure 15. In particular, the 90 µm powder addition resulted in a 7.1% increase for a 10 wt% fraction and a 30.2% increase for a 20 wt% fraction compared to the pure VE sample. A similar trend was observed with an increase to a 34 and a 33 MPa at the 20 wt% fraction for the 150 and 250 µm, respectively. This increase in strength was attributed to the presence of carbon fibers, of which the size is greater than that of the critical length, thus effectively transferring the load [34,35]. This was also affirmed by Heim et al. [36], who stated that for short fiber applications, the fiber’s critical length ($l_{c}$) needs to be twice that of the critical overlap length. The addition of a reinforcement more than 20% cause improper mixing of the reinforcement and an increase in the brittle behavior of the composite which results in a decrease in the tensile strength of the composite. An additional factor is the homogeneity of the carbon fibers within the matrix, adding up to the structure and further streamlining its ability to effectively transfer the load, which is reflected in the SEM images. With regards to the finely powdered carbon fibers (90 µm), Suresha et al. witnessed a similar trend where the powder uniform dispersion created better interfacial adhesion, which played a significant role in resisting the development of cracks in the vinyl ester matrix and hence improved the load-carrying capacity of the vinyl ester composite during tensile loading [37].

After the 20 wt% fraction, a downward trend was recorded of a value of 30.5 MPa for the 90 µm specimens while obtaining values of 24.8 MPa and 15.9 MPa for 150 µm and 250 µm, respectively, at the 40 wt% fraction. It is thought that the tensile strength reduction at higher reinforcement fractions is due to the non-uniform mixing between the powder/resin and the air entrapment (porosity) within the mixture [38,39,40]. In addition, this may be due to the decline in the net available short carbon fiber/vinyl ester interfacial area because of SCF accumulation or disputes in the dispersion of fiber bundles in the matrix [25]. This was also evident in the SEM images. With the increased fiber content, the decrease in strength could be attributed to the increased orientation randomness, where a similar trend was observed by Genna et al. [41].

**Figure 16:**
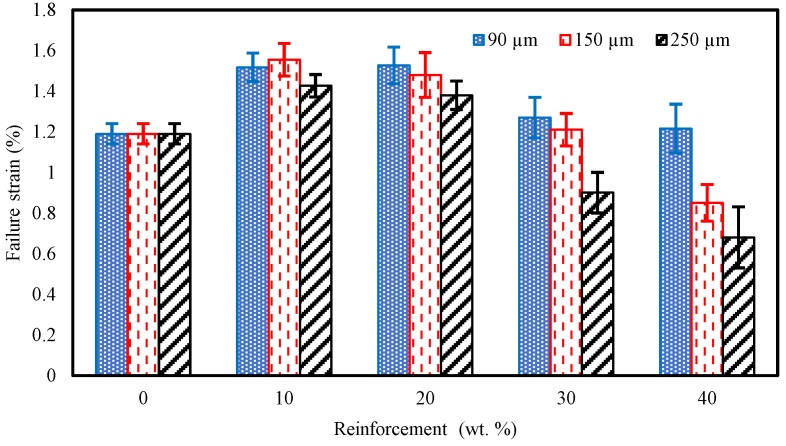
Tensile strain for carbon fiber-reinforced VE composites.

With the recycled composite tensile strain characteristics presented in Figure 16, it can be noted that a slight increase is obtained for the 90 µm samples up to the 20 wt% fraction with a respective value of 1.53%. A reduction followed this to 1.16% for the 40 wt% fraction which follows a similar trend as that of the tensile strength results in Figure 15. Regarding the 150 and 250 µm samples, the highest strain was recorded at a 10 wt% fraction and then decreased to 0.85% and 0.68%, respectively, at the 40 wt% fraction.

### 3.3. Compression Test

**Figure 17:**
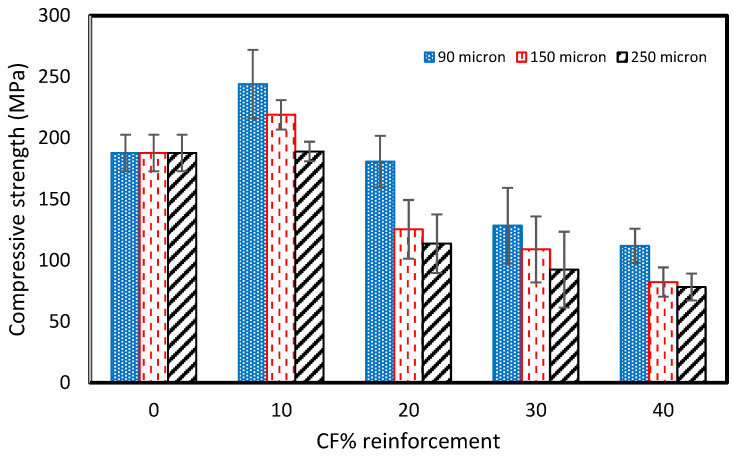
Compressive strength for carbon fiber reinforced VE composites.

A compression test was performed at room temperature (RT) on the prepared samples using the Instron Universal Testing Machine with a 50 kN load cell at a crosshead displacement of 5 mm/min. The compressive strength of the pure vinyl ester composite was recorded to be 187.75 MPa. The addition of reinforcement results in the increase in the compressive strength for 10 wt% of reinforcement. However, it was reduced with the further addition of recycled carbon powder to the composite. Figure 17 shows that the compressive strength increased by 30% from 187.5 MPa to 244 MPa with 10 wt% of the recycled carbon powder composite of a 90µm particle size. Samples prepared with 150 µm and 250 µm fiber size show approximately 17% and 1% increases in the compression strength with the addition of 10 wt% of recycled carbon powder. The compressive strength was reduced to 111.94 MPa, 82.2 MPa, and 78.23 MPa with 40% rCF for 90, 150, and 250 µm particle sizes. This decrease in the compressive strength indicates that the material produced became progressively more resistant to deformation. The increase in modulus further confirmed the loss of elasticity, which reflects increased rigidity. There are potential reasons that can explain the drop in the compression properties, such as the agglomeration of the reinforcement, which is also reflected in the SEM images. This results in poor compatibility [42,43]. In addition, the formation of hydrogen bonds between the filler content may cause the agglomeration of rCF [44].

### 3.4. Flexural

**Figure 18:**
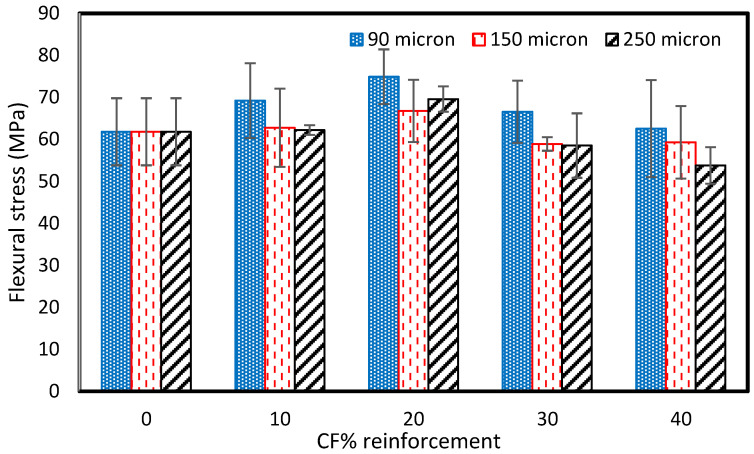
Flexural strength for carbon fiber-reinforced VE composites.

A flexural test was performed at room temperature (RT) on the prepared samples using an Instron Universal Testing Machine with a 50 kN load cell at a crosshead displacement of 1 mm/min. The flexural strength of the pure vinyl ester composite was 61.8 MPa. Figure 18 shows that the addition of reinforcement shows an increase in the flexural strength at first, followed by a decrease with further increments of the reinforcement. The results align with Li and Englund’s research [45]. The flexural strength was consistently higher with the addition of 10–40 wt% of 90µm rCFC than with the pure sample. However, it was lower than pure samples with additions of 30 and 40 wt% of 150 and 250 µm rCFC. The flexural strength was highest for the addition of 20% rCF of all fiber sizes. The addition of a reinforcement of more than 20% causes improper mixing and agglomeration of the reinforcement and consequently this may lead to more defects and high local stress concentration. This may also increase the brittle behavior of the composite which results a decrease in the flexural strength of the composite compared to the pure vinyl ester composites.

For 90 µm size particles, the flexural strength increased by 12% with the addition of 10 wt% rCF. The highest increase in the flexural was observed with 20 wt% rCFC, about 74.9 MPa. The flexural strength was 66.6 MPa and 62.6 MPa with 30 and 40 wt% rCFC, respectively. For 150 µm and 250 µm rCFC size particles, the highest flexural strength was observed with 20 wt% rCFC, which was about 66.8MPa and 69.6MPa, respectively. However, the lowest flexural strength was 59.3 and 53.8 for 150 µm and 250 µm rCFC size particles, respectively, obtained at 40 wt% rCFC reinforcement.

The scrap panels exhibited much higher values when compared to the recycled composite, which is attributed to the fact that the mechanically recycled carbon fibers are in discontinuous form and are not comparable to a continuous fiber composite. Furthermore, concerning the influence of the particle size on the mechanical properties of the recycled panels, it is evident that the smaller the particle size, the better the uniform distribution. It would possess during the fabrication of the samples, which would probably lead to better mechanical properties. A similar observation was noted by Gupta et al. [23], where the flexural properties were seen to improve with the addition of the powdered particles, which may be attributed to the improved surface area, thus allowing the interface/interphase to transfer the load effectively.

Further addition of reinforcement results in a decrease in mechanical properties, which indicates agglomeration and porosity in the prepared samples due to the non-uniform mixing of the reinforcement [42,43,44,45,46].

### 3.5. Thermal Conductivity

**Figure 19:**
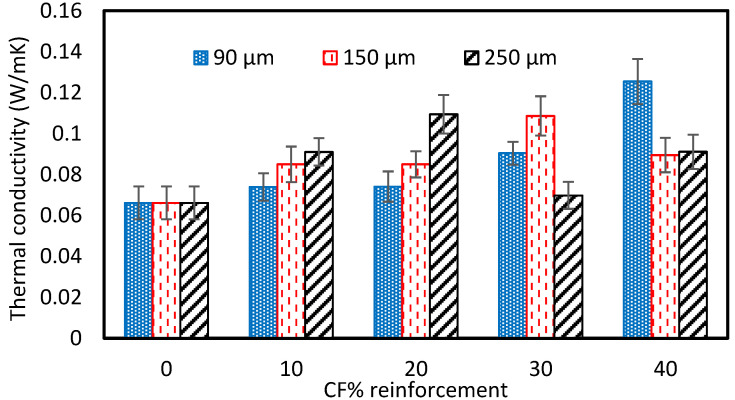
Thermal conductivity of carbon fiber-reinforced VE composites.

The thermal conductivity of the fabricated composite is shown in Figure 19. The thermal conductivity of the pure vinyl ester composite was 0.06615 W/mK. The thermal conductivity increased with the addition of reinforcement. It is due to the fact that the thermal conductivity of the carbon powder is higher than the pure vinyl ester resin. The thermal conductivity was 0.07387 W/mK, 0.8494 W/mK, and 0.09102 W/mK with 10 wt% for 90 µm, 150 µm, and 250 µm sizes of reinforcement, respectively, and was 0.0741 W/mK, 0.08494 W/mK, and 0.1094 W/mK for 20 wt% of reinforcement. With the further addition of the reinforcement, the thermal conductivity of the samples increased for 90 µm and reduced for 250 µm at 30 and 40 wt% of reinforcement. The maximum thermal conductivity value was 0.1254 for 90 µm with the addition of 40 wt% of reinforcement. The reduction in the thermal conductivity could be due to the voids developed due to the improper mixing during the fabrication of the samples [43]. A similar decrease in thermal conductivity with an increase in the weight percentage of the reinforcement was reported by Barkahd et al. [47].

### 3.6. Differential Scanning Calorimetry

**Figure 20:**
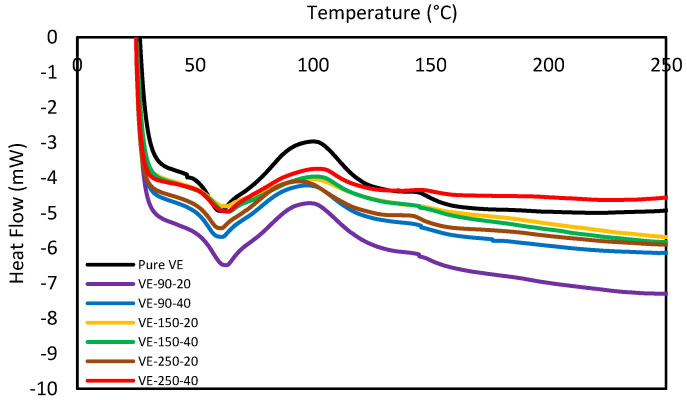
DSC of carbon fiber composites.

The DSC test was performed on the pure vinyl ester composite and recycled composite. Figure 20 presents the DSC curves for the samples prepared with reinforcement sizes of 90, 150, and 250 µm for the addition of 20 and 40 wt% of reinforcement. The DSC result shows that the pure vinyl ester composite sample’s glass transition temperature (Tg) was 52.92 °C. It was observed that the glass transition temperature was slightly increased with the addition of the reinforcement. However, the variation in the Tg of reinforced composing among themselves was very small. The Tg varied about 57 ± 1 °C for all combinations. For 20 wt%, the Tg was 56.9, 57.37, and 56.7 for 90, 150, and 250 µm, respectively. Furthermore, it was 57.857.9 and 56.6 for 90, 150, and 250 µm, respectively, for 40 wt% of the Tg.

In light of these results, two explanations are proposed for carbon fibers’ observed inhibition of vinyl ester polymerization. First, the carbon fiber reinforcements may inhibit vinyl ester polymerization through a phenomenon known as the ‘Trommsdorff’ or ‘gel’ effect [48]. By restricting the translational mobility of free radicals, the reinforcing fibers may aid radical trapping, depressing polymerization rates and the extent of the reaction [49]. Oxidative treatments may exaggerate the ‘Trommsdorff’ effect by increasing the surface roughness of the carbon fibers. Still, this effect alone is not sufficient to fully explain the additional inhibition induced via oxidation. It is not plausible that the increase in surface roughness from oxidative treatments would induce an inhibition equivalent to the fibers’ presence. A second explanation for the observed inhibition is that the carbon fiber surface chemistry interferes with vinyl ester free radical polymerization. An inhibitor of free radical polymerization is a species that scavenges propagating or initiator-derived radicals, preventing polymer chain formation. Inhibition occurs when a propagating free radical reacts with another species to produce an inactive polymer chain. Inhibition can also arise when a propagating radical reacts with another species to create a less reactive radical, a process termed retardation or degradative chain transfer [23,48,49].

### 3.7. Scanning Electron Microscopy

**Figure 21:**
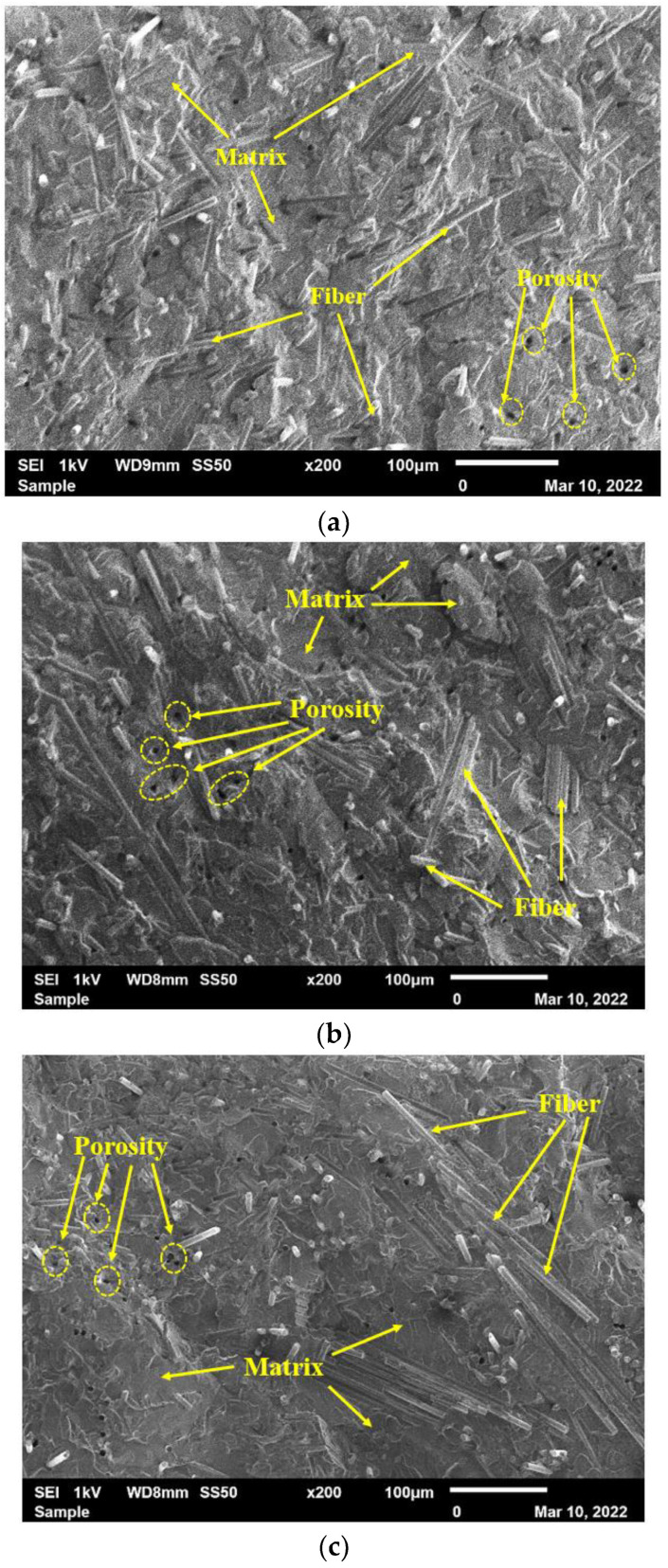
Fracture surfaces of samples fabricated with the addition of 20 wt% of recycled powder with various fiber sizes: (**a**) 90 µm, (**b**) 150 µm, and (**c**) 250 µm.

**Figure 22:**
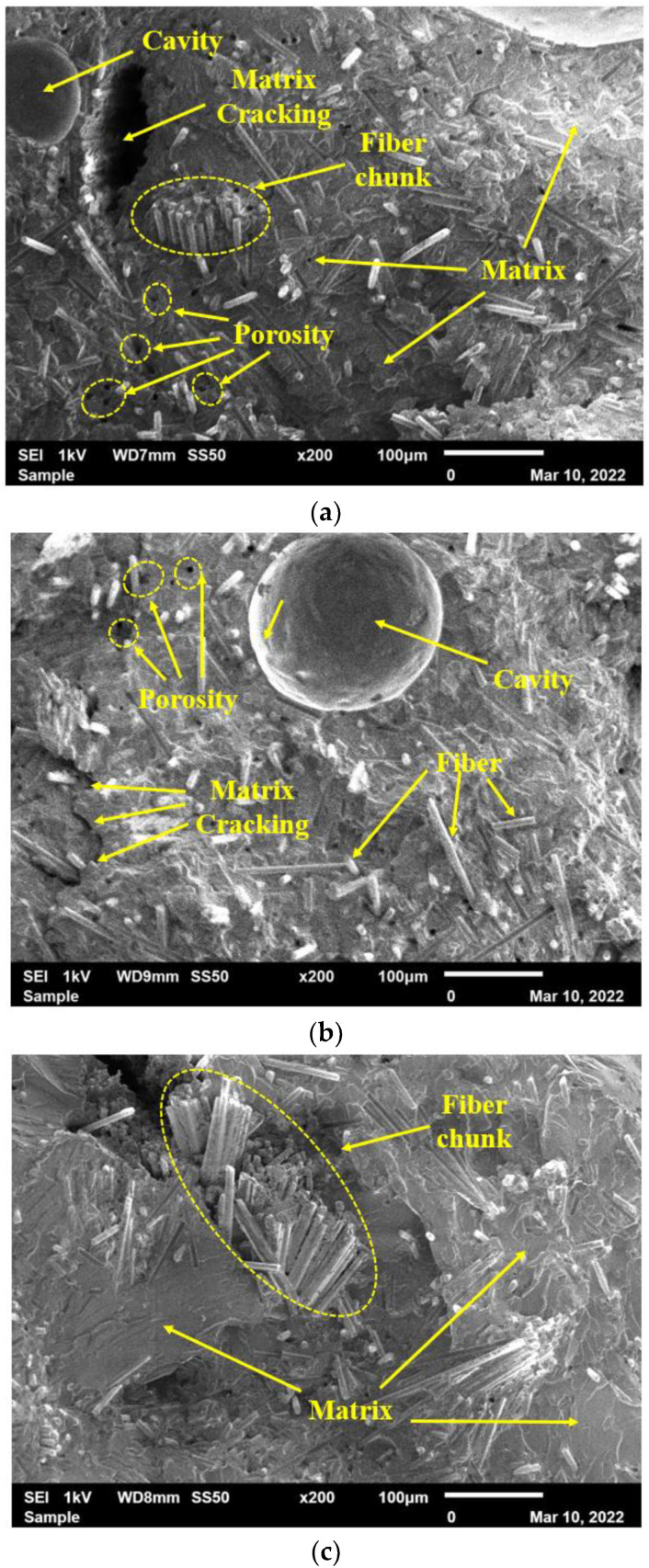
Fracture surfaces of samples fabricated with the addition of 40 wt% of recycled powder with various fiber sizes: (**a**) 90 µm, (**b**) 150 µm, and (**c**) 250 µm.

The fractured surfaces of tested samples were analyzed using a Scanning Electron Microscope (SEM). Figure 21 and Figure 22 represent the cross section of the fractured surfaces of samples fabricated with 20 and 40 wt% of reinforcement. It is clear from Figure 21 that reinforced fiber is uniformly mixed with the vinyl ester resin, resulting in improvements in the tensile and flexural properties of the composites. However, a small amount of porosity was also observed on the surface of the tested samples. It could be due to the small volume of gasses trapped inside the mixture during the plasticization, which developed due to the chemical reaction between the reinforcement and the vinyl ester resin.

Figure 22 shows the effect of further addition of reinforcement in the composite. The non-uniform distribution of reinforcement is prominent on the surface of the composite. The cavity on the surface of the composite shown in Figure 22a,b is mainly due to the air trapped during the mixing of the reinforcement in the resin. Also, the chunk of fiber observed on the surface of the composite, as shown in Figure 22a,c, results in reduced homogeneity across the cross-sectional area of the sample. Furthermore, the carbon fibers’ conglomeration minimizes the filler content’s surface area in contact with the matrix, resulting in weaker adhesion. This non-uniform distribution results in unexpected variations in the tensile and flexural properties of the composite.

The macrophotographs that were reported in Figure 21 and Figure 22 show several voids that are noticeable. The fracture morphology does not appear to be brittle and exhibits a fiber chunk/broom-like appearance, typical of extensive fiber–matrix debonding before fiber failure [50,51]. Cook and Gordon were the first to identify the mechanism by which longitudinal splitting is induced. In their book, the authors explain that assuming a tension crack propagates through the matrix in a unidirectional laminate, the shear stress generated at the fiber–matrix interface will be parallel to the fiber surface, eventually leading to a localized failure and inducing debonding of the fibers. However, it should be noted that this mechanism can also lessen the crack rate and inhibit further crack growth, where further fracture propagation will require a reinitiation of the crack [52]. The observation at a higher magnification confirmed the brooming of the fracture surface. It is also evident that the vinyl ester matrix adhesion is poor, where the fracture surface exhibits a lot of pulled-out fibers, and the exposed fibers are perfectly clean, implying a lack of resin grafting onto the rCFs [46]. These weak interfaces were not conducive to evenly distribute the load applied, leading to stress concentration and resulting in lower composite strength [25,53].

## 4. Conclusions

This study’s focus area was the recycling of carbon fiber composites obtained from the STRATA company. The cut-off/waste material produced during aircraft components manufacturing was mechanically reduced to powder form and used to prepare vinyl ester composites. Furthermore, fabricated composites were tested and analyzed to determine the composite’s various mechanical and physical properties. The major findings of the experimental testing are as follows:

- The tensile strength of the pure VE sample was 28.12 MPa. The cured carbon fiber powder addition to the vinyl ester (VE) resulted in a general tensile strength increase of up to 20 wt% and then a decrease with a further increase in reinforcement. The highest tensile strength was observed as 36.59 MPa for the composite fabricated with 20 Wt% of the 90 µm size reinforcement.
- The compressive strength of the pure vinyl ester composite was 187.75 MPa. The addition of reinforcement results in the increase in the compressive strength for 10 wt% of reinforcement. However, it was reduced with the further addition of recycled carbon powder to the composite. The compressive strength increased by 30% from 187.5 MPa to 244 MPa with 10 wt% recycled carbon powder composite of 90µm-sized fibers. The compressive strength was reduced to 111.94 MPa, 82.2 MPa, and 78.23 MPa with the addition of 40% rCFC for 90-, 150-, and 250 µm-sized fibers.
- The addition of reinforcement shows an increase in the flexural strength first, then decreases with a further increase in reinforcement. The flexural strength was consistently higher with the addition of 10–40 wt% of 90 µm rCFC than with the pure sample. However, it was lower than pure samples with additions of 30 and 40 wt% of 150 and 250µm rCFC. The flexural strength was highest for the addition of 20% rCFC of all fiber sizes.
- The thermal conductivity of the pure vinyl ester composite was 0.06615 W/mK. The thermal conductivity increased to 0.07387 W/mK, 0.8494 W/mK, and 0.09102 W/mK with 10 wt% for 90 µm, 150 µm, and 250 µm sizes of reinforcement, respectively. It was highest with a value of 0.1254 W/mK for 90 µm at the addition of 40 wt% of reinforcement, but reduced with the reinforcement of 150 µm- and 250 µm-sized fibers compared to the 20% wt value. The reduction in the thermal conductivity could be due to the voids developed due to the improper mixing during the fabrication of the samples.
- The fractured surfaces of tested samples were analyzed using a Scanning Electron Microscope (SEM). The reinforced fiber is uniformly mixed with the vinyl ester resin, which improves the tensile and flexural properties of the composites for 20 wt% of reinforcement. However, a small amount of porosity was also observed on the surface of the tested samples. The large cavities and fiber chunks were observed with the addition of 40 wt% of reinforcement. It is mainly due to the air trapped during the mixing of the reinforcement in the resin leading to unexpected variations in the tensile and flexural properties of the composites

## Figures and Tables

**Table 1:** Properties of Vinyl Ester resin.

Property	Unit	Value
Appearance	-	Clear liquid
Viscosity at 25 °C	cps	450–550
Color		Pinkish
Gel time at 25 °C	Min	30–50
Acid value KOH/g	mg	<14
Solid content	%	55 ± 2
Specific gravity at 25 °C	-	1.10–1.11
Peak temperature	°C	>170

**Table 2:** Specification of composite wastes.

Name	Resin Content (%)	Curing Class (C)	Fiber Areal Weight (g/m^2)
Cycom^®^977-2-35/40-12KHTS-134	35/40	180	134
Cycom^®^977-2-35/40-12KHTS-268	35/40	180	268

## Data Availability

Not applicable.
